# The Scottish and Irish Medical Schools

**Published:** 1907-09-07

**Authors:** 


### THE SCOTTISH AND IRISH MEDICAL SCHOOLS.

Differences of opinion doubtless exist as to the extent to which, in the interests of the whole, the principle of nationality should assert itself within the four corners of the United Kingdom. But no one would advocate its complete suppression, or would propose as a substitute a dull, drab uniformity. Some play and room for the development of individual characteristics and for the cultivation of local colour is to be desired, and particularly, perhaps, is this true in the field of education. The Scottish and Irish medical schools certainly help to discharge this function, and they can claim records of the most distinguished rank. Hence, apart from the interests of geographical convenience, it is gratifying to all who are anxious for the welfare of British medicine to know that these schools at the present day continue a line of policy which has brought to them a well-earned record of efficiency and success.

Of Scotland it has been said education is one of the staple products. Organised on a system particularly well adapted to meet the requirements of the country, her great Universities have for centuries ministered successfully to the educational wants of the people. They are essentially "corporations of students," and are in close and intimate contact with all ranks and classes of the community. These facts have largely influenced the teaching of medicine in Scotland. The various subjects included under this term have in a more or less complete fashion long constituted a "faculty" in each of the Universities, and as a consequence have been provided with an academic tone and atmosphere. The teaching has thus been framed on broad lines, and has been directed as much to the intellectual development of the student as to the provision for him of a technical equipment. Again, the medical students collected together in large Universities, and brought into close social contact with students of the other "faculties," have enjoyed the advantages which arise from such an association. In a word, the ideal of a Scottish medical education has always been the provision of a discipline which will develop the scientific spirit and will confer personal culture as well as technical efficiency. This ideal still remains. Note has been taken of the increasing demands of the scientific part of the curriculum, and by the help of the Carnegie Trust and the generosity of other donors, strenuous efforts have been made to meet these demands. Some of the recently constructed laboratories, indeed, may well fill with envy those whose lot has been cast in lands where no millionaire supports the policy of University development with the gift of a princely endowment. Altogether it may be confidently said that at no time in their long and glorious history have the medical faculties been in a higher state of efficiency and success. It must be remembered, too, that the fees are of moderate proportions, and the economical student, especially if he has the ability to win bursaries and scholarships, can accomplish the whole of the medical curriculum at a comparatively small expense. The Scottish people proverbially believe in education, and they insist that the highest academic opportunities shall be open to all those who have brains and energy to utilise them.

Educational efforts are not confined to the Universities in the narrow sense of the term. In Edinburgh, in Glasgow, in Aberdeen, and more recently at Dundee in connection with St. Andrews, the large hospitals are practically the clinical schools of the University, and the clinical teaching is organised at a very high and efficient level. It is, and indeed always has been, a feature of Scottish medical education that the clinical instruction shall be arranged on a definite method and system equally with the courses of systematic lectures. The Scottish Universities have now not a few imitators south of the Tweed, and in the generous rivalry which needs must exist, we have every confidence they will prove themselves equal to their great reputation.

Medicine in Ireland can claim many great names in physic, and many influential clinical teachers. Here, as in Scotland, it has long enjoyed a secure position as a subject of academic study, and Irish medical graduates have in many instances attained to the highest rank and position in the public services of the Empire. There is no question of the efficiency of the teaching in the Irish schools. It has always, it may be said, been distinguished by a decidedly practical note, and by a very high standard of professional obligation and duty. The contributions made from these schools to the progress of clinical medicine and clinical surgery are the common heritage of the profession. In the University of Dublin, and the Royal University of Ireland, there are thoroughly organised arrangements for teaching all departments of the medical curriculum, and the degrees of these Universities are universally recognised as of high value and distinction. The Royal Colleges, again, grant licenses to practise, and also, through the agency of their higher diplomas, encourage advanced work in medicine and surgery. The reputation of the various Dublin hospitals as efficient clinical schools is universally allowed, whilst for the provinces successful colleges and medical schools exist at Belfast, Cork, and Galway. Altogether the Irish medical student has first-rate opportunities, and he may legitimately be proud of any distinctions which he may gain in the educational institutions of his native land.

